# Multi-Objective and Parallel Particle Swarm Optimization Algorithm for Container-Based Microservice Scheduling

**DOI:** 10.3390/s21186212

**Published:** 2021-09-16

**Authors:** Xinying Chen, Siyi Xiao

**Affiliations:** School of Software, Dalian Jiaotong University, Dalian 116000, China; xiaosiyi1997@163.com

**Keywords:** multi-objective optimization, container-based microservice scheduling, particle swarm optimization algorithm, cloud computing

## Abstract

An application based on a microservice architecture with a set of independent, fine-grained modular services is desirable, due to its low management cost, simple deployment, and high portability. This type of container technology has been widely used in cloud computing. Several methods have been applied to container-based microservice scheduling, but they come with significant disadvantages, such as high network transmission overhead, ineffective load balancing, and low service reliability. In order to overcome these disadvantages, in this study, we present a multi-objective optimization problem for container-based microservice scheduling. Our approach is based on the particle swarm optimization algorithm, combined parallel computing, and Pareto-optimal theory. The particle swarm optimization algorithm has fast convergence speed, fewer parameters, and many other advantages. First, we detail the various resources of the physical nodes, cluster, local load balancing, failure rate, and other aspects. Then, we discuss our improvement with respect to the relevant parameters. Second, we create a multi-objective optimization model and use a multi-objective optimization parallel particle swarm optimization algorithm for container-based microservice scheduling (MOPPSO-CMS). This algorithm is based on user needs and can effectively balance the performance of the cluster. After comparative experiments, we found that the algorithm can achieve good results, in terms of load balancing, network transmission overhead, and optimization speed.

## 1. Introduction

In recent years, microservices have become increasingly popular as a new application development model and have been widely used in cloud computing. An application based on the microservice architecture is designed as a set of independent, fine-grained modular services. Each service separately performs various tasks and uses a lightweight communication mechanism to transfer information between different microservices. Each user’s needs can be addressed through a group of collaborative microservices. Due to the advantages of using containers in cloud architecture, such as limited management costs, easier and faster deployment, and higher portability, the use of containers within cloud architectures has become widespread, as is the case for Netflix [1], Amazon [2], IBM [3], Uber [4], and Alibaba [5].

Application containerization is one of many technologies that helps to create microservice architectures [6]. Containerization is a method used to realize the virtualization of an operating system. Container, as a lightweight virtualization technology based on the operating system layer, provides a separate execution environment and file system to run applications. Containers use a sandbox mechanism and, as a result, there will be no interfaces between containers, almost no overhead, and they can easily be run in a data center. The most important advantage is that it does not depend on any language, framework, or system; therefore, the Docker Container instance can greatly reduce the cost of virtualization. Compared with a virtual machine, Docker Container has less consumption, is simpler, and can be deployed faster. Current mainstream container management tools include Docker Swarm [7], Apache Mesos [8], and Google Kubernetes [9]. Despite the rapid development of these technologies and a certain number of practical container-based microservice scheduling solutions, there are still some important issues that need to be resolved in container-based microservice scheduling.

Three scheduling strategies are commonly used in the currently popular container cluster management tool Docker Swarm [10]: Spread, Binpack, and Random. In the Kubernetes scheduler, there are two: the predicate phase and the priority phase. These two management tools only focus on the use of physical resources, ignoring other aspects such as network overhead and cluster load balancing. An effective scheduling scheme should be more comprehensive, such that the allocation of computing resources and storage resources of physical nodes is more effective. While realizing cluster load balancing, local load balancing should also be realized. To achieve this, comprehensive consideration of service reliability and network transmission overhead is required. Further research is needed to create such a scheduling method.

The container-based microservice scheduling problem is a typical NP-hard problem. At present, many researchers use many methods to solve the virtual machine scheduling problem in cloud computing. Daniel Guimaraes Lago et al. [11] have proposed a container-based microservice scheduling algorithm based on resource type awareness. This algorithm includes two parts: The first finds the optimal deployment of physical machines for the container, and the other reduces the network transmission power consumption. Mosong Zhou et al. [12] have inferred task resource requirements based on similar task runtime information and proposed a fine-grained resource scheduling method. Carlos Guerrero et al. [13] have proposed a genetic algorithm approach with the aim of finding a suitable solution to address the problem of container allocation and elasticity using the NSGA-II. Lin Miao et al. [14] have proposed a multi-objective optimization model for container-based microservice scheduling with the aim of solving the scheduling problem using an ant colony algorithm. Nguyen Dinh Nguyen et al. [15] aimed to overcome the bottleneck problem through use of a leader election algorithm, which functions by evenly distributing the leaders throughout the nodes in a cluster. Salman Taherizadeh et al. [16] considered the specific quality of service (QoS) trade-offs and proposed an innovative capillary computing architecture.

These methods can solve the container-based microservice scheduling problem, to some extent; however, most of them can only achieve cluster load balancing, and cannot achieve local load balancing. These methods are prone to uneven use of resources within the node, resulting in unreasonable container allocation, which leads to increased transmission overhead and reduced reliability. At the same time, these methods suffer from slow optimization speeds and can easily fall into local optima. In order to solve these problems, we first re-design the representation of the scheduling scheme. Then, in order to reduce transmission overhead, improve cluster reliability, and load balancing, three target models are proposed. Finally, a parallel particle swarm optimization algorithm [17] is used, in order to solve the multi-objective optimization problem of container-based microservice scheduling.

The main contributions of this paper are as follows.

- First, we establish three new optimization target models for the container-based microservice scheduling problem: the network transmission cost model between microservices, the global and local load balancing model, and the service reliability model. The optimization target model proposed in this paper can solve the above-mentioned problems that exist in current methods, at least to a certain extent.
- Second, a new representation of the scheduling scheme and particle is proposed to increase the searching speed. Based on this representation, a new particle swarm updating method is proposed, which preserves the diversity of particles while also approaching the optimal solution during the optimization process.
- Finally, a parallel particle swarm optimization algorithm is used to solve the multi-objective optimization problem of container-based microservice scheduling. The algorithm utilizes Pareto-optimal theory to select the individual extremum and the global extremum of the particle swarm to improve the optimization efficiency of the algorithm. At the same time, parallel computing is used to improve the solution speed of the algorithm. Through inter-process communication, particle swarms can exchange optimal solutions with each other, thus improving the efficiency of the global search and allowing the particle swarms to avoid falling into local optima.

The rest of this paper is structured as follows. Section 2 briefly introduces related technologies. Section 3 proposes the three optimization objective models. Section 4 introduces the multi-objective optimization parallel particle swarm optimization algorithm for container-based microservice scheduling (MOPPSO-CMS) in detail. Section 5 provides the experimental comparison and analysis, and concludes the paper.

## 2. Related Technologies

This section introduces the techniques and theories used in this paper.

### 2.1. Particle Swarm Optimization

Particle swarm optimization (PSO) was first proposed by Eberhart and Kennedy in 1995 [18]. Its basic concept was derived from study of the foraging behavior of birds. The PSO algorithm was inspired by the behavioral characteristics of biological populations and can be used to solve optimization problems. The standard PSO algorithm is detailed in the following equations:

(1) $$V_{i}\left(k+1\right)=\omega \times V_{i}\left(k\right)+c_{1}\times rand\left(\right)\times \left(pbest_{i}-X_{i}\right)+c_{2}\times rand\left(\right)\times \left(gbest-X_{i}\right),$$

(2) $$X_{i}\left(k+1\right)=X_{i}\left(k\right)+V_{i}\left(k+1\right),$$

where ω is the inertia factor, and $c_{1}$ and $c_{2}$ are learning factors, representing their own inertia and the influence of individual extrema and the global extremum on particles, respectively. The vector $X_{i}=\left\{x_{i1},x_{i2},\cdots ,x_{iN}\right\}$ represents the position of particle *i* in the *N*-dimensional search space. $V_{i}=\left\{v_{i1},v_{i2},\cdots ,v_{iN}\right\}$ represents the velocity of particle *i*. Each particle can be regarded as a search unit in the *N*-dimensional search space. Particles update themselves through two extreme values: The first is their personal best position (shown as $pbest_{i}$), while the other is the best position found by the whole population (shown as gbest). The particles will always update their positions according to these two extreme values, until the optimal solution is found.

**Figure 1:**
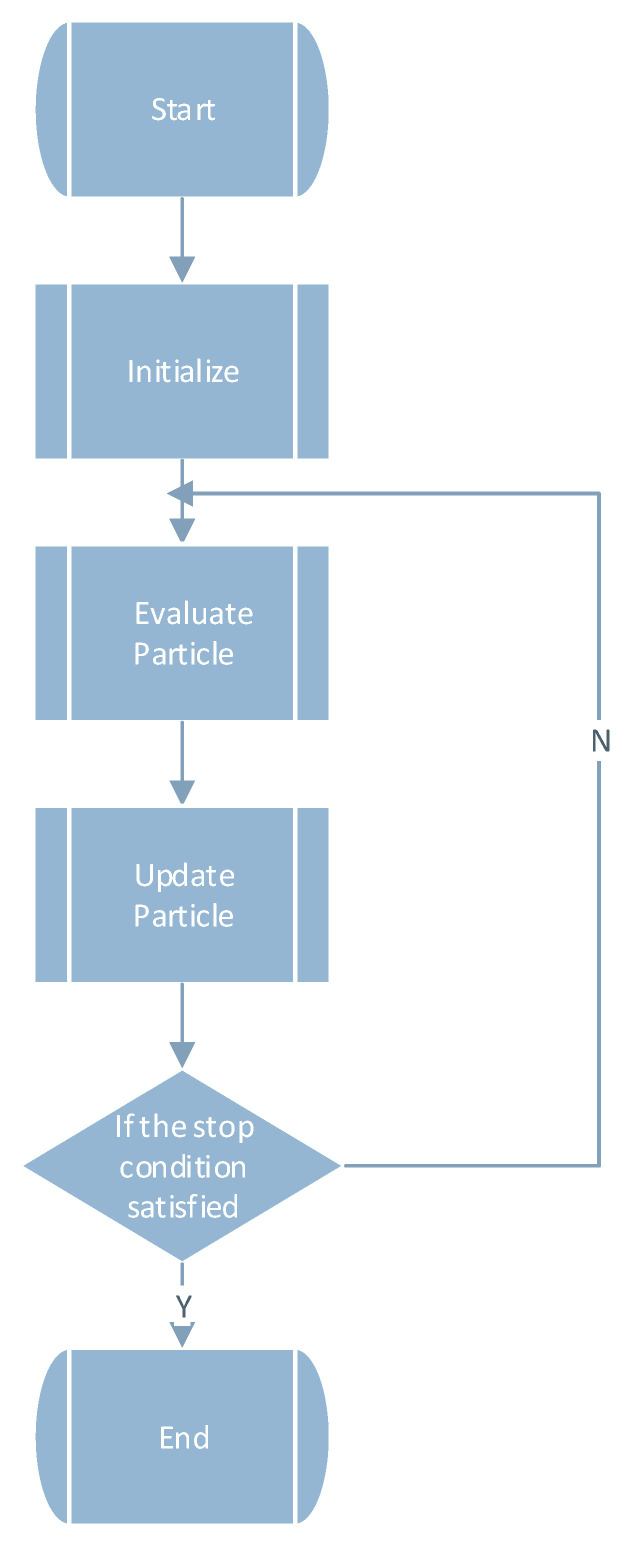
Flow chart of the particle swarm optimization algorithm.

The particle swarm optimization algorithm has been widely used in various fields and has many efficient variations. Jun Sun et al. [19] proposed the quantum particle swarm optimization algorithm, which combines the PSO algorithm with quantum behavior to solve the traditional problems of the PSO algorithm. Lifeng Xu et al. [20] proposed a hybrid particle swarm optimization algorithm with multi-level disturbance to prevent the PSO algorithm from falling into local optima. Liu K. et al. [21] proposed a Bayesian network structure optimization method based on local information, by adding the PSO algorithm. Lingxia Liao et al. [22] aimed to solve a generic controller placement problem (GCP) by planning the placement of controllers over SDN systems; to achieve this, they proposed a novel multi-objective genetic algorithm (MOGA) with a mutation based on a variant of the PSO algorithm. Muhammad Salman Qamar et al. [23] aimed to settle the traveling salesman problem (TSP) by proposing a novel best–worst ant system (BWAS) based on the PSO algorithm. Xianjia Wang et al. [24] investigated the role of the particle swarm optimization (PSO) strategy update rules in the evolution of cooperation in the prisoner’s dilemma (PD) on scale-free networks. The flow chart of the particle swarm optimization algorithm is shown in Figure 1.

The pseudocode of the particle swarm optimization algorithm is shown in Algorithm 1.
**Algorithm 1:** Particle swarm optimization algorithm
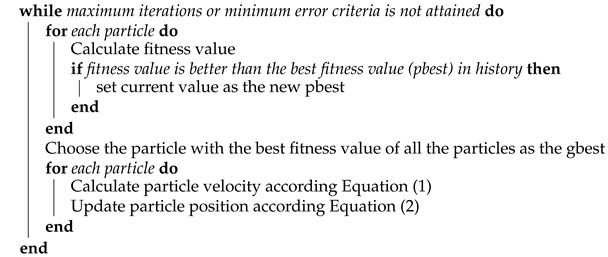


### 2.2. Pareto Optimality Theory

Pareto optimality is an ideal state of resource allocation, assuming that an inherent group of people and allocated resources change from one allocated state to another, making at least one person better without making anyone worse; this is referred to as Pareto improvement or Pareto optimization. The following section introduces some important concepts behind Pareto optimality.

1. Pareto Dominance
  For the objective function $f\left(x\right)=[f_{1}\left(x\right),\cdots ,f_{n}\left(x\right)]$, if solution $\vec{x}=\left(x_{1},\cdots ,x_{m}\right)$ can Pareto-dominate solution $\vec{v}=\left(v_{1},\cdots ,v_{m}\right)$, it must satisfy $\forall f_{i}\left(x\right)⩽f_{i}\left(v\right)\wedge \exists f_{j}\left(x\right)<f_{j}\left(v\right),i,j\in \left(1,\cdots ,n\right)$. If and only if the objective function value of any solution $\vec{x}$ is not greater than that of solution $\vec{v}$, and there exists at least one objective function value less than that of value $\vec{v}$, we say that solution $\vec{x}$ Pareto-dominates solution $\vec{v}$.
2. Pareto Non-inferior Solutions
  For the objective function $f\left(x\right)=[f_{1}\left(x\right),\cdots ,f_{n}\left(x\right)]$, if solution $\vec{x}=\left(x_{1},\cdots ,x_{m}\right)$ is Pareto non-inferior to solution $\vec{v}=\left(v_{1},\cdots ,v_{m}\right)$, it must satisfy $\exists f_{i}\left(x\right)<f_{i}\left(v\right)\wedge \exists f_{j}\left(x\right)>f_{j}\left(v\right),i,j\in \left(1,\cdots ,n\right)$. If and only if solution $\vec{x}$ is better than solution $\vec{v}$ on some objective functions, and solution $\vec{x}$ is worse than solution $\vec{v}$ on some objective functions, then solution $\vec{x}$ is not inferior to solution $\vec{v}$.
3. Pareto Optimal Solution
  For the objective function $f\left(x\right)=[f_{1}\left(x\right),\cdots ,f_{n}\left(x\right)]$, if there is no solution in the solution set $X=[{\vec{x}}_{1},\cdots ,{\vec{x}}_{k}]$ that can Pareto-dominate solution ${\vec{x}}_{p},p\in \left(1,\cdots ,k\right)$, then the solution ${\vec{x}}_{p}$ is the Pareto-optimal solution. Multi-objective optimization problems usually have many Pareto-optimal solutions, and the solution set of Pareto-optimal solutions is called the Pareto-optimal front.

Pareto-optimal theory has been used to solve multi-objective optimization problems in different fields. Divya Chhibber et al. [25] aimed to obtain the Pareto-optimal solution of a multi-objective fixed-charge solid transportation problem, by using the unique approach of intuitionistic fuzzy programming with linear, hyperbolic, and exponential membership, as well as non-membership functions. Srinivas Nagaballi et al. [26] utilized a game theory-based (minimax) algorithm to take the best decision from a set of non-dominated solutions obtained by Pareto optimality criteria. Marcin Czajkowski et al. [27] discussed a multi-objective evolutionary approach to the induction of model trees, in order to demonstrate how a set of non-dominated model trees can be obtained using a global model tree (GMT) system.

Many researchers have combined particle swarm optimization with Pareto optimization to solve multi-objective optimization problems. Hu Wang et al. [28] optimized the Pareto entropy through use of the target space transformation method, and introduced the concepts of lattice dominance and lattice distance density, in order to evaluate the fitness of the Pareto-optimal solution. On this basis, a multi-objective particle swarm optimization algorithm based on Pareto entropy was formed. Yuliang Shi [29] proposed a service pricing model based on Pareto-optimality and used the particle swarm optimization algorithm to find the optimal service pricing and resource allocation strategy. Chang’an Shi et al. [30] proposed a shared aperture dynamic allocation method based on environmental information for a radar-communication-integrated radio frequency system, combining Pareto-optimal theory and an improved particle swarm optimization algorithm based on integer coding.

## 3. Multi-Objective Optimization Model

This section first introduces the system model and then puts forward three new optimization target models and a new multi-objective optimization model. The names and descriptions of relevant parameters are summarized in Table 1.

### 3.1. System Model

An application based on a container-based microservice architecture can be represented as a tuple <MS_SET,MS_RELATION> [13], where MS_SET is the set of microservices of the application and MS_RELATION is the set of consumption relationships between the microservices of the application. If a microservice completes a task and needs to use the result of other microservices, there is a consumption relationship between the two microservices. This relationship can be defined as $(ms_{cons},ms_{prov})\in MS\_RELATION$, where $ms_{cons}$ represents the consumer and $ms_{prov}$ represents the provider.

A microservice $ms_{i}$ can be represented as a tuple $<Calc\_Req_{i},Str\_Req_{i},Mem\_Req_{i},Fail_{i},CONS\_SET_{i},Link\_Thr_{i},Scale_{i}>$ [13], where $Calc\_Req_{i}$ is the computing resources required by a container of the microservice $ms_{i}$, $Str\_Req_{i}$ is the storage resource required by a container of the microservice $ms_{i}$, and $Mem\_Req_{i}$ is the memory resource required by a container of the microservice $ms_{i}$. $Fail_{i}$ is the failure rate of microservice $ms_{i}$. $CONS\_SET_{i}$ is a set of microservices that is consumed by microservice $ms_{i}$; that is, if $(ms_{i},ms_{l})\in MS\_RELATION$, then $ms_{l}\in CONS\_SET_{i}$. $Link\_Thr_{i}$ is the upper limit of the request that can be processed by a single container instance of microservice $ms_{i}$. $Scale_{i}$ is the number of containers for microservice $ms_{i}$, each container corresponding to a microservice, and a microservice can have multiple container instances.

As mentioned above, there is a consumption relationship between microservices and microservices, and containers and containers. They transfer requests, where the number of requests from microservice $ms_{i}$ to microservice $ms_{l}$ can be represented as $Link(ms_{i},ms_{l})$. The amount of data required for a request between microservice $ms_{i}$ and microservice $ms_{l}$ is expressed as $Trans(ms_{i},ms_{l})$. When the sender is a user or a client, the amount of data transmitted is not considered; we only consider the number of requests in this paper.

A physical node, $pm_{j}$, can be represented as a tuple $<Calc\_Res_{j},Str\_Res_{j},Mem\_Res_{j},$$Fail_{j}>$. Each microservice can deploy one or more containers on any physical node, where $Calc\_Res_{j}$ is the calculating resource that physical node $pm_{j}$ can provide, $Str\_Res_{j}$ is the storage resource that physical node $pm_{j}$ can provide, and $Mem\_Res_{j}$ is the memory resource that physical node $pm_{j}$ can provide. The total consumption of containers on a single physical node cannot exceed that provided by the physical node. Physical nodes may cause downtime, denial of service, or computational exceptions due to software or hardware problems; as such, $Fail_{j}$ represents the physical node failure rate [31]. Physical nodes are connected through the network, and the network distance between each physical node is expressed as $Dist(pm_{j},pm_{j^{\prime }})$. $PassTime(cont_{i},cont_{k})$ represents the time required for data transmission between two containers, and the closer the containers are, the shorter $Dist(pm_{j},pm_{j^{\prime }})$ and $PassTime(cont_{i},cont_{k})$ are.

**Figure 2:**
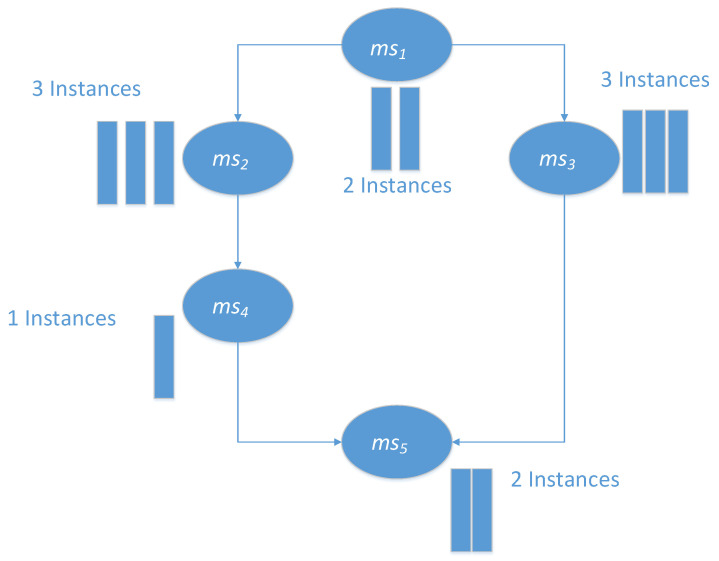
A simple application example.

A simple application is shown in Figure 2. This graph shows a directed acyclic graph (DAG) of Job-A. There are five different microservices, corresponding to the five different microservices in the graph. In the execution process, microservice1, microservice2, microservice3, microservice4, and microservice5 have 2, 3, 3, 1, and 2 instances, respectively. The execution of microservice2 and microservice3 depends on the completion of microservice1, while the execution of microservice5 depends on the completion of microservice4 and microservice3 [32].

### 3.2. Multi-Objective Optimization Model

In this section, we introduce the problems present in the models in [13,14], then propose three new target models. The authors of [14] proposed a container-based microservice scheduling ant colony multi-objective optimization algorithm (ACO-MCMS); while the authors of [13] proposed a container-based microservice scheduling genetic multi-objective optimization algorithm (GA-MOCA). These algorithms have large network transmission costs, unbalanced clusters and individual loads, and long optimization times.

In order to reduce the transmission overhead, provide load balancing, and improve service reliability and algorithm operation efficiency between microservices, we designed three new target models, completely redesigning the representation of the scheduling scheme, as detailed in the following sections. For a detailed explanation of the equation, please refer to the works in [13,14].

#### 3.2.1. Network Transmission

The model of network transmission overhead in ACO-CMS [14] is defined as follows:

(3) $$\begin{array}{c} COMM\left(x\right)=\sum_{j=1}^{n}\sum_{i=1}^{m}\frac{x_{i,j}}{Scale_{i}}\sum_{l=1\wedge l\neq j}^{n}\sum_{ms_{k}\in CONS\_SET_{i}}\frac{x_{k,l}}{Scale_{k}}\times \\ Link\left(ms_{i},ms_{k}\right)Trans\left(ms_{i},ms_{k}\right)Dist\left(pm_{j},pm_{l}\right), \end{array}$$

where $x_{i,j}$ represents whether the container of microservice *i* is allocated to physical node *j*. If the container of microservice *i* is allocated to physical node *j*, then $x_{i,j}=1$; otherwise, $x_{i,j}=0$. In any physical node, there can be at most one container instance from the same microservice [14]. This model uses the average network distance of all the container pairs between consumer and provider microservices to calculate the data transmission overhead between two microservice containers.

The model of network transmission overhead in GA-MOCA [13] is defined as follows:

(4) $$\begin{array}{c} TotalNetworkDistance=\sum_{\forall ms_{i}}ServiceMeanDistance\left(ms_{i}\right), \end{array}$$

(5) $$\begin{array}{c} ServiceMeanDistance(ms_{i})=\\ \frac{\sum_{\forall cont_{k}|cont_{k}\equiv ms_{i}}\left(\sum_{\forall cont_{k^{\prime }}\equiv ms_{i^{\prime }}|{\left(ms_{i^{\prime }},ms_{i}\right)}_{prov/cons}}dist_{alloc\left(cont_{k}\right),alloc\left(cont_{k^{\prime }}\right)}\right)}{|cont_{k}|\times |cont_{k^{\prime }}|}, \end{array}$$

where $cont_{k}^{\prime }\equiv ms_{i}$ means that container $cont_{k}$ encapsulates/executes microservice $ms_{i}$, $alloc\left(cont_{k}/ms_{i}\right)=pm_{j}$ means that the physical machine $pm_{j}$ allocates service $ms_{i}$/container $cont_{k}$, and $|cont_{k}|$ is the total number of containers. This model approximates the network overhead between the microservices of an application using the average network distance between all the pairs of consumer and provider containers.

In the GA-MOCA algorithm model, only the network distance between physical nodes is considered, while the request and transmission amounts are ignored. In the ACO-CMS algorithm model, although considering the request quantity and transmission quantity on the basis of GA-MOCA, there are still shortcomings. In the process of network transmission, there are obvious differences in transmission speed, distance, and other factors between containers allocated in the same physical node and containers allocated in different physical nodes. This problem is not adequately solved in the models of the above two papers; therefore, we propose a new network transmission overhead model definition:

(6) $$\begin{array}{c} Trans\_Consume\left(type\right)=Link\left(cont_{i},cont_{k}^{type}\right)Trans\left(cont_{i},cont_{k}^{type}\right)\\ Dist\left(cont_{i},cont_{k}^{type}\right)PassTime\left(cont_{i},cont_{k}^{type}\right), \end{array}$$

(7) $$\begin{array}{c} Inner\_Consume=\frac{\sum_{i=1}^{n}\sum_{k\in CONS\_SET}Trans\_Consume\left(in\right)}{Scale_{i}}, \end{array}$$

(8) $$Outer\_Consume=\frac{\sum_{i=1}^{n}\sum_{k\in CONS\_SET}Trans\_Consume\left(out\right)}{Scale_{i}},$$

(9) Total_Consume=Inner_Consume+Outer_Consume.

The total network transmission overhead, Total_Consume, consists of the network transmission overhead between containers assigned to the same physical node Inner_Consume and the network transmission overhead between containers assigned to different physical nodes Outer_Consume. Trans_Consume(type) indicates the calculation method of network transmission consumption under different types. According to type, the $cont_{k}^{type}$ is divided into $cont_{k}^{in}$ and $cont_{k}^{out}$. $cont_{i}$ represents the container instance of microservice $ms_{i}$. Container instances of microservices that have consumer relationships with microservice $ms_{i}$ and are assigned to different physical nodes, represented as $cont_{k}^{out}$. Containers of microservices that have consumer relationships with microservice $ms_{i}$ and are assigned to same physical nodes are represented as $cont_{k}^{in}$. Based on the GA-MOCA algorithm and ACO-CMS algorithm models, this model focuses on the difference between the network overhead transmitted between the containers of the same physical node and the network overhead allocated between the containers of different physical nodes. Considering the transmission time issues, the optimization of the transmission overhead is more comprehensive.

#### 3.2.2. Load Balancing

The model of load balancing in ACO-CMS [14] is defined as

(10) $$\begin{array}{c} RESRC\_CONS\left(X\right)=\frac{1}{\sigma_{1}+\sigma_{2}}\max_{1\leq j\leq n}\max\\ (\sum_{i=1}^{m}x_{i,j}\frac{Link_{i}\times Cal\_Reqst_{i}}{Scale_{i}\times Cal\_Resrc_{i}}\sigma_{1},\sum_{i=1}^{m}x_{i,j}\frac{Link_{i}\times Str\_Reqst_{i}}{Scale_{i}\times Str\_Resrc_{i}}\sigma_{2}), \end{array}$$

where $Cal\_Reqst_{i}$, $Cal\_Resrc_{i}$, $Str\_Reqst_{i}$, and $Str\_Resrc_{i}$ have the same meaning as $Cal\_Req_{i}$, $Cal\_Res_{i}$, $Str\_Req_{i}$, and $Str\_Res_{i}$ in this paper, respectively. $\sigma_{1}$ and $\sigma_{2}$ are the standard deviation values of the utilization rate of computing resources and storage resources of the physical nodes in the cluster, respectively. This model operates on the assumption that the worst load of the cluster is not necessarily the maximum resource utilization rate with a relatively balanced resource load, but a high resource utilization rate with a relatively unbalanced resource load.

The model of load balancing in GA-MOCA is defined as

(11) $$\begin{array}{c} BalanceClusterUse=\sigma \left(PM_{usage}^{pm_{l}}\right),if\;\;\exists ms_{i}|alloc\left(ms_{i}\right)=pm_{l}, \end{array}$$

(12) $$PM_{usage}^{pm_{l}}=\frac{\sum_{ms_{i}}\frac{ureq_{i}\times msreq_{i}\times res_{i}}{scale_{i}}}{cap_{l}},\forall ms_{i}|alloc\left(ms_{i}\right)=pm_{l},$$

(13) $$ThresholdDistance=\sum_{\forall ms_{i}}\left|\frac{ureq_{i}\times msreq_{i}\times res_{i}}{scale_{i}}-thr_{i}\right|,$$

where $ureq_{i}$ denotes the number of user requests for application *i*, $msreq_{i}$ denotes the number of microservice requests $ms_{i}$ needed for each $ureq_{j}$ request from application *j*, and $res_{i}$ denotes the computational resources required for a microservice request. In this mode, we define a metric called the threshold distance, which is the difference between the resource consumption of a container and the threshold value of a microservice. This is formalized in Equation (13), which uses the standard deviation of the percentage of resource usages of the physical nodes, in order to evaluate the balance of the cluster.

It is obvious that GA-MOCA ignores other factors relevant to load balancing. On the basis of GA-MOCA, the influence of storage on load balancing is added to the ACO-CMS model. Using the maximum value of the resource utilization rate with the coefficient among the physical nodes reflects the worst-case load for the load balancing of the cluster. Although the use of a maximum value is more comprehensive, it ignores the combined effects of other factors on load balancing. This will lead to inefficiency in storage and computational resources.

The models of these two papers cannot adequately address these problems. In order to address these problems, we propose a global load balancing approach in this paper. Global load balancing consists of cluster load balancing, which is the load balancing of the whole physical node cluster, and local load balancing, which means the resources are balanced within one physical node. Global load balancing aims to achieve the load balancing of the entire physical node cluster and the rational use of the entire cluster resources at the same time. The objective model of load balancing is designed as follows:

(14) $$CalcStrDif_{j}=\left|\frac{Calc\_Req_{j}}{Calc\_Res_{j}}-\frac{Str\_Req_{j}}{Str\_Res_{j}}\right|,$$

(15) $$StrMemDif_{j}=\left|\frac{Str\_Req_{j}}{Str\_Res_{j}}-\frac{Mem\_Req_{j}}{Mem\_Res_{j}}\right|,$$

(16) $$MemCalcDif_{j}=\left|\frac{Mem\_Req_{j}}{Mem\_Res_{j}}-\frac{Calc\_Req_{j}}{Calc\_Res_{j}}\right|,$$

(17) $$\begin{array}{c} LocalLoadBalancing=\frac{\sum_{j=1}^{n}\left(CalcStrDif_{j}+StrMemDif_{j}+MemCalcDif_{j}\right)}{3n}, \end{array}$$

(18) $$ClusterLoadBalancing=\frac{\sigma_{clac}+\sigma_{str}+\sigma_{mem}}{3},$$

(19) $$\begin{array}{c} GlobalLoadBalancing=\frac{ClusterLoadBalancing+LocalLoadBalancing}{2}, \end{array}$$

where LocalLoadBalancing is the sum of the differences of the ratio between the three resources of the physical node. The differences are represented as CalcStrDif, StrMemDif, and MemCalcDif. The larger the value is, the more unbalanced it is. $\sigma_{calc}$, $\sigma_{str}$, and $\sigma_{mem}$ represent the standard deviation of computing resources, storage resources, and memory resources used throughout the physical node cluster, respectively. ClusterLoadBalancing is calculated using three standard deviations. The greater the standard deviation, the more discrete and the more unbalanced the use. GlobalLoadBalancing is the mean of ClusterLoadBalancing and LocalLoadBalancing. We intend to achieve cluster load balancing for each resource, making each resource use more reasonable. In this paper, the physical node storage, memory, and computing resources are calculated, combining the local load balancing with cluster load balancing.

#### 3.2.3. Service Reliability

The reliability model in ACO-CMS [13] is defined as follows:

(20) $$Link\_Fail\left(x\right)=\sum_{j=1}^{n}\sum_{i=1}^{m}Fail_{j}\times x_{i,j}\frac{Link_{i}}{Scale_{i}}.$$

This model uses the average number of request failures as an indicator to measure the reliability of cluster services, which is mainly related to the number of microservice requests and the failure rate of the nodes.

The reliability model in GA-MOCA is defined as

(21) $$ServiceFailure\left(ms_{i}\right)=\prod_{\forall pm_{l}|allocation\left(ms_{i}\right)=pm_{l}}\left(fail_{l}+\prod_{\forall ms_{i}|allocation\left(ms_{i}\right)=pm_{l}}fail_{i}\right).$$

This model measures the reliability of the system through the failure rate of the applications. An application fails when any of its microservices fail, and a microservice fails when all of the container replicas fail. A container fail is generated by a fail in the container, $fail_{i}$, or by a fail in the physical machine that allocates the container, $fail_{l}$.

As both physical nodes and containers may have unpredictable errors due to various problems, the number of requests failed is an important indicator to measure the reliability of a service. The definition of the model in GA-MOCA is multiplicative. When the number of microservices and physical nodes is large, the result is too small, which is not conducive to calculation by the computer and the comparison between the results. Compared with GA-MOCA, the failure rate of physical nodes is only considered in the ACO-CMS model, while the failure rate of containers is ignored. In addition, the container instance of the same microservice in each node in the ACO-CMS model is unique. This constraint means that the ACO-CMS model is unable to find an effective allocation scheme in the case of more container instances and less physical nodes. To solve the above problems, the model proposed in this paper is as follows:

(22) $$InnerFail=\frac{Link(cont_{i},cont_{k}^{in})}{Scale_{k}}\left(fail_{i}+fail_{k}\right),$$

(23) $$OuterFail=\frac{Link(cont_{i},cont_{k}^{out})}{Scale_{k}}\left[fail_{j}+\left(1-fail_{j}\right)\left(fail_{i}+fail_{k}\right)\right],$$

**Figure 3:**
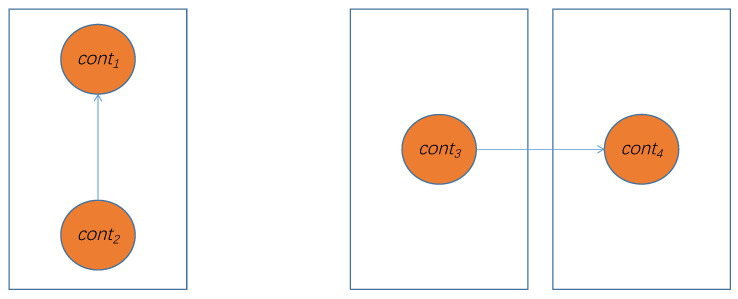
A simple container transfer example.

(24) SystemFail=InnerFail+OuterFail,

where InnerFail refers to the number of requests that may fail when transmitting between containers in the same physical node, and OuterFail refers to the number of requests that may fail when transmitting between containers in different physical nodes. Requests sent between containers within the same physical node are only affected by container failure rates; however, sending requests between containers of different physical nodes is affected not only by the failure rate of the container itself, as represented by $fail_{i}$ and $fail_{k}$, but also by the failure rate of the physical node, $fail_{j}$. As shown in Figure 3, $cont_{1}$ and $cont_{2}$ are assigned to same physical node, such that the fail rate only depends on the containers; however, $cont_{3}$ and $cont_{4}$ need to transfer data from physical node to physical node, so the fail rate not only depends on the containers, but also on the physical nodes. The model in this paper calculates the number of failure requests that may occur in the container transmission within the same node and between different nodes to solve this problem—the model can even reasonably calculate and compare when there are multiple containers and multiple physical nodes.

### 3.3. Multi-Objective Optimization Model

Under physical node resource constraints, a multi-objective optimization model based on the above three models is established for the container-based microservice scheduling problem.

(25) $$\begin{array}{c} minimize\;Total\_Consume(x) \end{array}$$

(26) $$\begin{array}{c} minimize\;LoadBalancing(x) \end{array}$$

(27) $$\begin{array}{c} minimize\;SystemFail(x) \end{array}$$

(28) $$\begin{array}{c} s.t.\;Calc\_Req_{j}\leq Calc\_Res_{j} \end{array}$$

(29) $$\begin{array}{c} s.t.\;Str\_Req_{j}\leq Str\_Res_{j} \end{array}$$

(30) $$\begin{array}{c} s.t.\;Mem\_Req_{j}\leq Mem\_Res_{j} \end{array}$$

Functions (25)–(27), respectively, represent the three optimization objectives: minimizing network transmission overhead, rationalizing load balancing, and minimizing the number of requests failed. Functions (28)–(30) represent the computing resource constraints, storage resource constraints, and memory resource constraints of the physical node, respectively. The resources used by the container on the physical node cannot outnumber the resources available to the physical node.

It is difficult to solve multi-objective optimization problems directly, especially to find the optimal solution. Particle swarm optimization algorithms have been widely used in various problems and have achieved respectable results. Pareto theory is a decent framework that can be used to deal with multi-objective optimization problems; therefore, we use an algorithm that combines the particle swarm optimization algorithm and the Pareto frontier, using Pareto theory to evaluate the quality of the function solution. Through the global extremum, individual extremum, self-inertia, and the interaction between multiple particle swarm groups, our algorithm can avoid falling into local optima and, thus, ensure the quality of the solution.

## 4. Parallel Particle Swarm Optimization Algorithm

Traditional particle swarm optimization easily converges to local optima during the optimization process [33]. We use a parallel particle swarm optimization algorithm, in order to address this problem. Based on the traditional PSO algorithm, MOPPSO-CMS increases the number of simultaneous iterative particle swarms and allows the particle swarms to communicate with each other through inter-process communication, exchanging the optimal solution to avoid falling into local optima.

### 4.1. Representation of Particles and Scheduling Scheme

**Figure 4:**
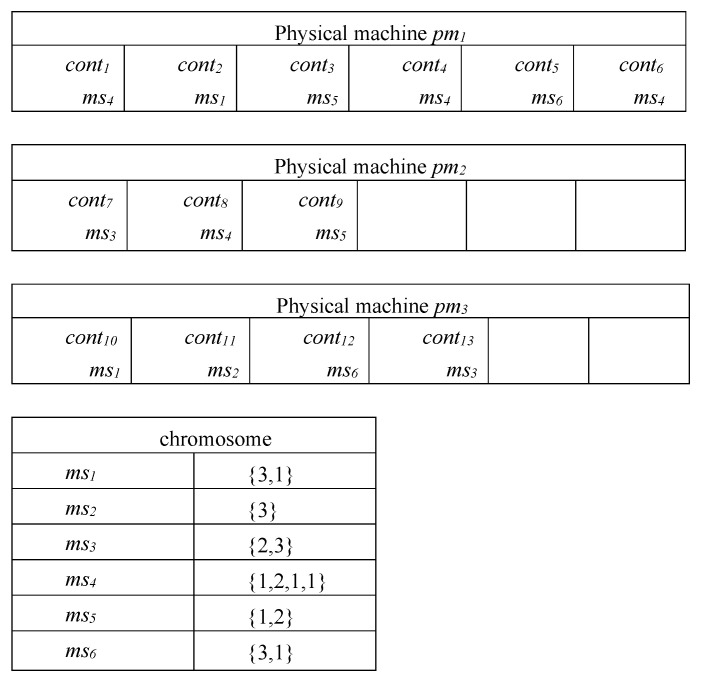
Representation of chromosome and scheduling scheme in GA-MOCA.

In the study of GA-MOCA and ACO-CMS, solutions for this problem are usually based on a string-based notation. For example, GA-MOCA defined a string-based notation to represent the number of containers for each microservice, as well as the allocation of these containers to the physical machines, as shown in Figure 4.

After investigating this method, we found that it has several problems: First, according to the characteristics of the ACO-CMS algorithm, when it tries to find a suitable schedule scheme, it has to traverse each container, microservice, and physical node separately, which results in significant search times. If there are *x* containers, *y* microservices, and *z* physical nodes, and the ACO-CMS algorithm has a population of *m* particles and *n* iterations, the time complexity of the ACO-CMS algorithm is O(x×y×z×m×n).

Second, in the GA-MOCA algorithm, when the crossover and mutation operations occur, unreasonable solutions are always generated (i.e., growth mutation, swap mutation, and shrink mutation). Growth mutation adds a physical node to a microservice randomly, for example, if $ms_{6}=\left\{3,1\right\}$, then perhaps $ms_{6}=\left\{3,1,1\right\}$ after mutation. Swap mutation exchanges the allocation of microservices, for example, if $ms_{6}=\left\{3,1\right\}$ and $ms_{2}=\left\{3\right\}$, then $ms_{2}=\left\{3,1\right\}$ and $ms_{6}=\left\{3\right\}$ after mutation. Shrink mutation reduces the physical node that the microservice has been allocated, for example, if $ms_{6}=\left\{3,1\right\}$, then perhaps $ms_{6}=\left\{3\right\}$ after mutation. Therefore, if $ms_{6}$ only has two container instances when the operations occur, $ms_{6}$ may not fill the quantity limit or exceed the resources that the physical nodes can provide. This can generate an invalid schedule scheme. This is the case for all of the other operations, as well.

Third, when there are large amounts of containers and microservices, the representation method uses significant amounts of memory to record the allocation order of containers when the algorithm is running, and the allocation order of containers has no direct impact on the optimization of the scheduling plan; however, this is suitable for the operation of their algorithm, specifically.

**Figure 5:**
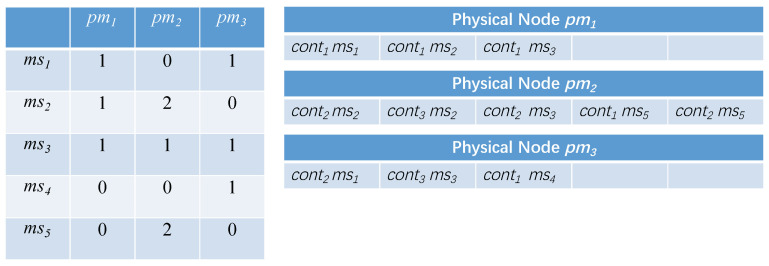
The new scheduling scheme and particle representation.

Considering the above problems, we define a new scheduling scheme expression, based on the number of containers. Each scheduling scheme is represented by a two-dimensional array, each row representing a microservice $ms_{i}$, and each column represents a physical node $pm_{j}$. The element $\left(ms_{i},pm_{j}\right)$ represents the number of containers of microservice $ms_{i}$ allocated to physical node $pm_{j}$. Consider the simple application we mentioned above (shown in Figure 2) as an example; one of its schedule schemes (or particles) is shown in Figure 5. Figure 5 shows the original state of the particle, which is randomly initialized by the MOPPSO-CMS algorithm. As microservice1 has two container instances, the total number of rows in $ms_{1}$ is two. The allocations $(ms_{1},pm_{1})=1$ and $(ms_{1},pm_{3})=1$ are randomly initialized, where one of the $ms_{1}$ containers is assigned to $pm_{1}$ and the other is assigned to $pm_{3}$. Compared to the previous representation method, this method has several advantages:

First, the new representation method and the characteristics of MOPPSO-CMS algorithm have reduced time complexity. When the MOPPSO-CMS algorithm begins, it first initializes the particles (shown in Figure 5), then finds the suitable schedule scheme by changing the number of containers in the physical node, instead of traversing each container and physical node. Thus, if there are *x* containers, *y* microservices, and *z* physical nodes, and the MOPPSO-CMS algorithm has a population of *m* particles and *n* iterations, the time complexity of the MOPPSO-CMS algorithm is O(y×m×n).

Second, the transfer and copy operations, which are discussed later, can avoid generating an invalid schedule scheme while looking for a suitable schedule scheme, as they do not change the total number of containers.

Third, the memory resource of the new representation method only depends on the number of microservices and the physical nodes. The amount of containers will not significantly affect the new representation method.

In conclusion, the new representation method combines the advantages and overcomes the shortcomings of both ACO-CMS and GA-MOCA. ACO-CMS will not generate an invalid schedule scheme, as it picks the containers in order to find suitable physical nodes; however, this may result in increased time complexity. The GA-MOCA may have less time complexity, but can generate many invalid schedule schemes. The new representation will reduce the time complexity and avoid generating invalid schedule schemes at the same time, thus combining the advantages of both methods.

### 4.2. Transfer and Copy Operations

**Figure 6:**
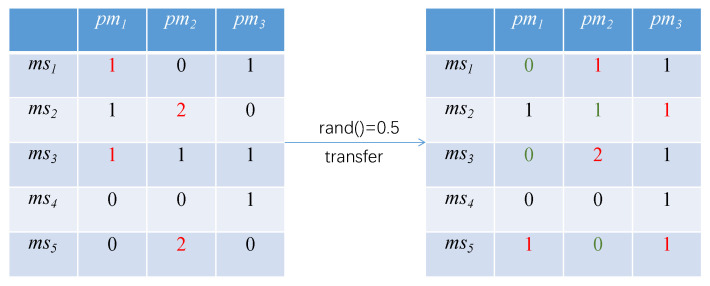
Transfer operation of particles.

The original update method of the PSO [18] is shown in Equations (1) and (2). Obviously, the original update method of the particle swarm does not apply to the algorithm in this paper. To solve this problem, we improve the update method based on the original. The first is the transfer operation. In order to ensure the optimization ability of the particle itself, each particle is transferred according to a probability; namely, the inertia factor ω. The transfer operation of particles is illustrated in Figure 6.

In the figure, there is a 0.5 probability for the transfer operation to occur in each position of the particle. If the transfer occurs, the microservice would randomly transfer its containers to other physical nodes. For example, if a transfer occurs at $\left(ms_{1},pm_{1}\right)$, the containers in the physical node $pm_{1}$ are randomly transferred to $pm_{2}$. Similarly, if a transfer occurs at $\left(ms_{5},pm_{2}\right)$, the containers in the physical node $pm_{2}$ are randomly transferred to $pm_{1}$ and $pm_{3}$. The number of the transfer containers are random, for example, for $\left(ms_{2},pm_{2}\right)$, it could transfer one or two to $pm_{3}$. If the number of containers in the position is 0, no transfer occurs.

**Figure 7:**
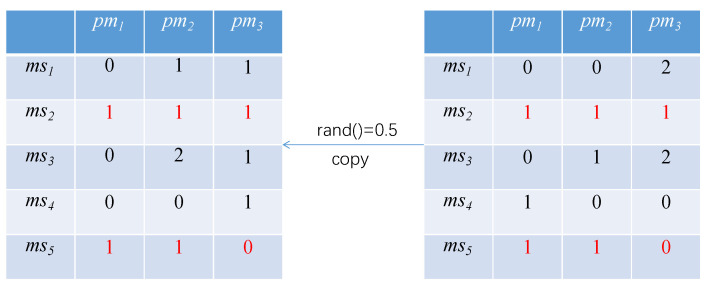
Copy operation of particles.

Further, in order to increase the global optimization ability and optimization efficiency, the copy operation is integrated into the process of particle swarm optimization. Each row in the particle will copy the individual extremum pbest and the global extremum gbest according to a specified probability (i.e., the learning factors $c_{1}$ and $c_{2}$), taking the particle itself and the individual extremum pbest as an example. The copy operation of the particle is illustrated in Figure 7.

In the figure, the left side is the particle, and the right side is the individual extremum pbest of the particle. According to the learning factor, the probability of a copy operation occurring is 0.5. The copy operation occurs at $ms_{2}$, and the particle copies the elements of the same row in pbest, covering their own elements to achieve the purpose of learning from the individual extremum.

### 4.3. Parallel Particle Swarm Optimization Algorithm

The traditional PSO algorithm only uses one swarm when running; in contrast, the parallel particle swarm optimization algorithm in this paper uses multiple swarms operating at the same time. First, the MOPPSO-CMS algorithm is used to initialize the particles, as shown in Figure 5. Then, the algorithm calculates gbest and pbest, according to the fitness function. The fitnesses of the particles are defined as an array (Total_Consume,GlobalLoadBalancing,SystemFail); the quality of the particles is assessed by means of an objective function of optimization problems [34]; and each element of the array is calculated using Equations (9), (19) and (24), respectively. These three equations represent the fitness function used in our method. The smaller the fitness, the better the particle.

In the MOPPSO-CMS algorithm, each swarm has their own gbest, pbest, and Pareto-optimal front. Within the swarm, after initializing, the particle is updated through the transfer and copy operations mentioned above. First, according to ω, it executes the transfer operation; the containers allocated are transferred to other physical nodes. Second, the particle copies the rows from pbest, according to $c_{1}$, to execute the copy operation. Third, the particle copies the rows from gbest, according to $c_{2}$, to execute the copy operation.

When the particle is initialized or changed, its fitness is calculated. According to Pareto optimality theory, if the new fitness (which, in our approach, is named $pbest^{\prime }$) Pareto-dominates pbest, then pbest is replaced by $pbest^{\prime }$, and the schedule scheme of pbest is also replaced by the schedule scheme of $pbest^{\prime }$. Otherwise, we keep pbest and the associated schedule scheme. Then, the gbest or Pareto-optimal front is updated to the same operation.

When the iteration is finished, the fitness of each particle is compared to that of the others. One with fitness that is Pareto-dominated by another particle will be dropped. The rest is the gbest, and forms the Pareto-optimal front. It is difficult to find the best solution in a multi-objective optimization problem. Therefore, the gbest is not unique in this algorithm. According to the Pareto optimality theory mentioned above, each Pareto-optimal solution is a gbest, and the set of gbest (or Pareto-optimal solutions) is a Pareto-optimal front.

Each iteration generates a new set of gbest, with the gbest in the new set denoted by $gbest^{\prime }$. The $gbest^{\prime }$ are compared with gbest in the Pareto-optimal front. All gbest that are Pareto-dominated by the $gbest^{\prime }$ are dropped, and the $gbest^{\prime }$ are added to the Pareto-optimal front. If any $gbest^{\prime }$ is Pareto-dominated by any one of the gbest, then it is dropped. If $gbest^{\prime }$ does not Pareto-dominate any gbest, and all gbest do not Pareto-dominate $gbest^{\prime }$, then $gbest^{\prime }$ is added to the Pareto-optimal front.

**Figure 8:**
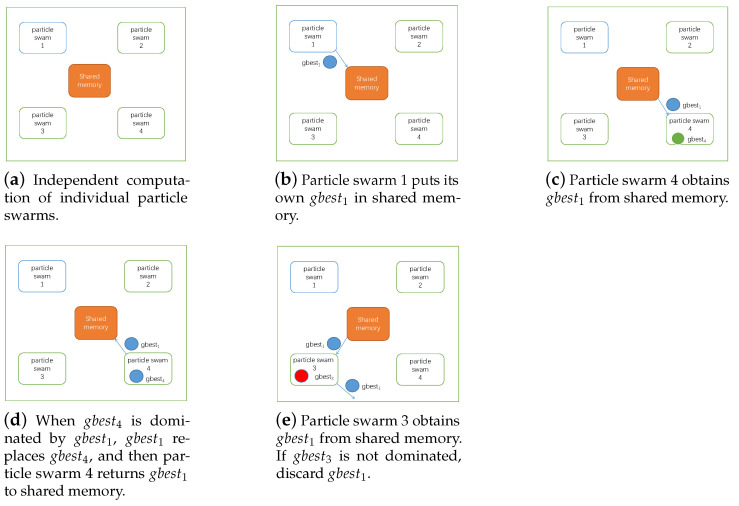
Flow of the inter-process communication.

When the Pareto-optimal front of the swarm is updated, inter-process communication is carried out. The swarm uploads the $gbest^{\prime }$ that was added most recently to the Pareto-optimal front and the shared memory. The other swarm downloads the $gbest^{\prime }$ from the shared memory, all the local gbest Pareto-dominated by the $gbest^{\prime }$ are dropped, and the $gbest^{\prime }$ are added to the Pareto-optimal front. The $gbest^{\prime }$ are uploaded to the shared memory again, for the rest of the swarm to download. If the $gbest^{\prime }$ is Pareto-dominated by any one of the local gbest, then the $gbest^{\prime }$ is dropped. If $gbest^{\prime }$ does not Pareto-dominate any gbest, and all gbest do not Pareto-dominate $gbest^{\prime }$, then the $gbest^{\prime }$ is added to Pareto-optimal front, and the $gbest^{\prime }$ is uploaded again. The operation of inter-process communication is shown in Figure 8.

The particle or schedule scheme is output, which has the minimum value of a sum of fitness in the Pareto-optimal front. The algorithm pseudo-code is shown in Algorithm 2.
**Algorithm 2:** Parallel particle swarm optimization algorithm
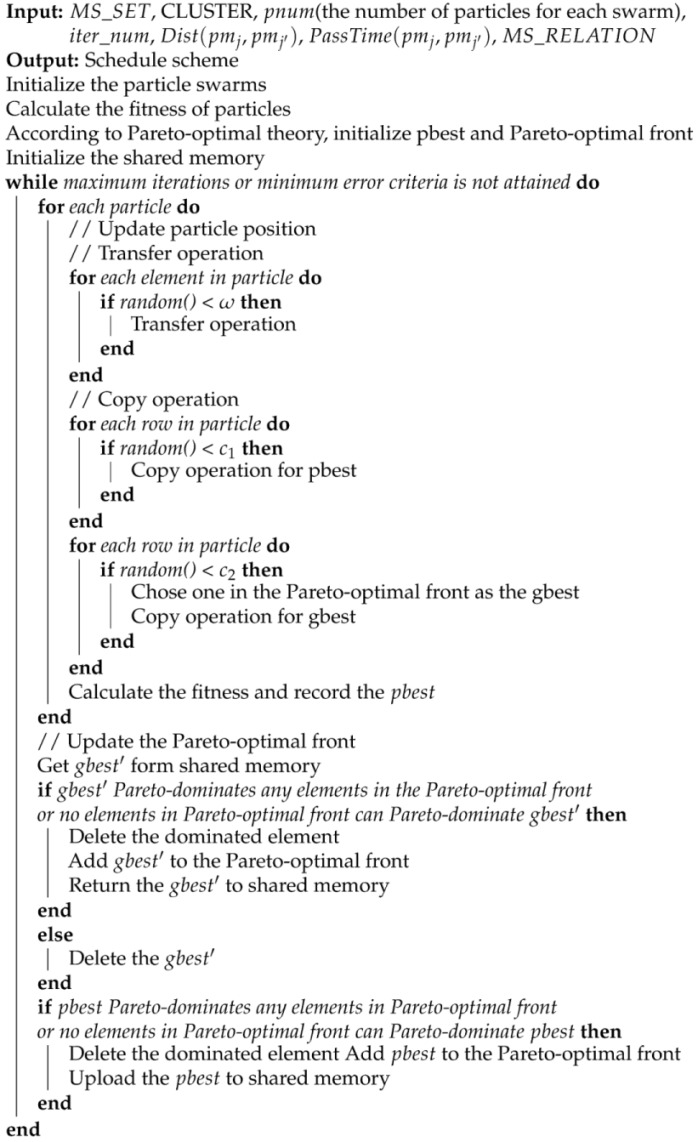


## 5. Experiment and Analysis

### 5.1. Experimental Data

We conducted experiments based on the Alibaba Cluster Trace V2018 cluster [30] data set. Cluster-trace-v2018 released a new version of cluster tracking in 2018 as part of the Alibaba Open Cluster Tracking Program. The data set contains approximately 4000 computers containing eight days of information, composed of the following six tables:

1. machine_meta.csv: Machine metadata and event information.
2. machine_usage.csv: Resource usage of each machine.
3. container_ eta.csv: Meta-information and event information of containers.
4. container_usage.csv: Resource usage for each container.
5. batch_instance.csv: Information about instances in batch workload.
6. batch_ task.csv: Information about instances in batch workload.

The experimental test data set is shown in Table 2 [13] and Table 3. Table 2 shows the consumption relationship $\left(ms_{i},ms_{l}\right)$, the amount of data transmission $Trans_{i,j}$, and the number of connections $Link_{i,j}$ between microservices when the application receives a user request. If the microservice is an entry microservice, for the first started microservice in the application, the consumption relationship is $\left(0,ms_{i}\right)$. Table 3 shows the relevant values of the microservices to complete a user request, where CONS_SET, $Cal\_Req_{i}$, $Str\_Req_{i}$, and $Mem\_Req_{i}$ are abbreviated as CONS, $Cal_{i}$, $Str_{i}$, and $Mem_{i}$, respectively. The explanation of each parameter is referenced in Table 1.

### 5.2. Algorithm Parameters Settings

The algorithm parameters are designed as follows:

(1) Number of physical nodes: |CLUSTER| = 100.
(2) Physical nodes have three different computing capabilities: $Calc\_Res_{j}=\left[100,200,400\right]$.
(3) Physical nodes have three different storage capabilities: $Str\_Res_{j}=\left[100,200,400\right]$.
(4) Physical nodes have three different memory capabilities: $Mem\_Res_{j}=\left[60,120,180\right]$.
(5) The failure rate of physical nodes is a random number from 0.01 to 0.03.
(6) The network distance between container transmissions $Dist(pm_{j},pm_{j^{\prime }})$, the same physical node is 1, and there are four different physical nodes.
(7) When the time required to transfer data between containers is the same as the physical node PassTime=1; when the node is different PassTime=4.

Table 4 shows the minimum of the sum of fitness for the MOPPSO-CMS algorithm under different parameters. The best results of the experiment have been shown in bold. From Table 4, we can see that the growth of iter_num, $c_{2}$, and ω improved the algorithm. Most fitness values have been reduced, and $c_{2}$ has a significant impact on the algorithm. Further, we noticed that when $c_{1}$ grows, it negatively impacts the performance of the algorithm. $c_{1}$ indicates the possibility of the particle to copy rows from pbest, the growth of $c_{1}$ may cause the algorithm to fall into local optima. ω indicates the possibility of a particle to perform a transfer operation, where the growth of ω may improve the global-optimization ability of the algorithm. $c_{2}$ indicates the probability of a particle to copy rows from gbest, where properly increasing the value can improve the optimization ability of the algorithm; however, if the value is too high, it will lead to the algorithm falling into local optima. When considering the effects of every parameter, we increased the value of $c_{2}$, ω, pnum, and iter_num and reduced the value of $c_{1}$. We can see that, when parameters were $pnum=300,iter\_num=300,c_{1}=0.1,c_{2}=0.45,\omega =0.45$, the algorithm obtained the best performance; therefore, we chose these as the parameters of the algorithm. The parameters of our algorithm are shown in Table 5.

### 5.3. Related Algorithms for Comparison

In this paper, we conducted our experiment on a Windows 10 system. The processor was an Intel Core i7-8750H, the memory was 16 GB, the display card was an NVIDIA RTX 2070, and the language used was Python. In order to verify the effectiveness of the MOPPSO-CMS algorithm, we compared it with the ACO-CMS [14], GA-MOCA [13], and Spread [16] algorithms.

The ACO-CMS algorithm is a container-based multi-objective optimization algorithm used for microservice scheduling. This algorithm considers the utilization of computing resources and storage resources of physical nodes, the number of requests, the failure rate of physical nodes, and combines multi-objective heuristic information to improve the efficiency of optimal path selection.

The GA-MOCA algorithm is a multi-objective optimization algorithm used for container microservice scheduling, based on NSGA-II. This algorithm considers the threshold distance of the container, load balancing of the physical node computing resources, and the reliability of the service and communication overhead between related microservices.

Spread is a scheduling strategy owned by Docker Swarm, which selects the physical nodes with the least container instances for deployment.

### 5.4. Experimental Results and Analysis

We compared the performance of the four algorithms considering six different aspects: network transmission overhead, local load balancing, standard deviation of cluster resources, global load balancing, service reliability, and algorithm running speed.

#### 5.4.1. Network Transmission Overhead

**Figure 9:**
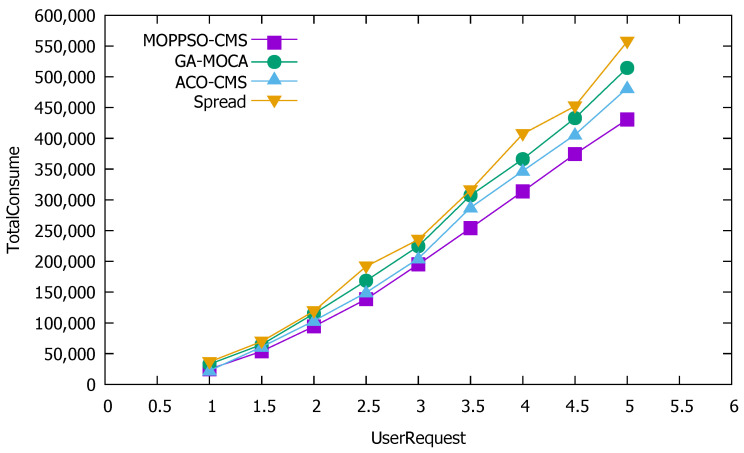
Performance differences in network transmission overhead among the four considered algorithms.

Network transmission takes into account the number of requests, transmission data size, transmission distance, transmission time, and other factors in the process of network transmission, and so network transmission overhead is one of the indicators we used to measure the performance of the algorithm. The smaller its value, the less overhead the network transmission requires. Figure 9 shows the performance differences in network transmission overhead among the four algorithms.

The Spread algorithm selects a physical node with the least container instances for deployment each time. The algorithm distributes the microservice containers as evenly as possible to each physical node, leading to nodes with consumption relations that are easily assigned to different physical nodes. Thus, the transmission overhead between microservices is greatly increased.

GA-MOCA considers the influence of the distance between physical nodes in finding solutions, so its effect is slightly better than Spread.

ACO-CMS optimizes factors such as physical node distance and transmission data size, but ignores the possible impact of transmission time. Moreover, due to the characteristics of the ACO-CMS algorithm, the container instance of the microservice on a physical node is unique, so it is impossible to deploy multiple consumption-related microservice containers to the same physical node.

Given the significant difference in the number of two microservice containers, the result was an increase in network transmission overhead. Figure 9 shows that the Spread algorithm performed the worst, and it always had the highest network transmission overhead, with the ACO-CMS and GA-MOCA algorithms performing better; however, the MOPPSO-CMS algorithm achieved the best performance, as it considers the influence of the data transmission amount and network distance on the network transmission overhead, as well as the influence of time required for data transmission on the network transmission overhead.

#### 5.4.2. Local Load Balancing

**Figure 10:**
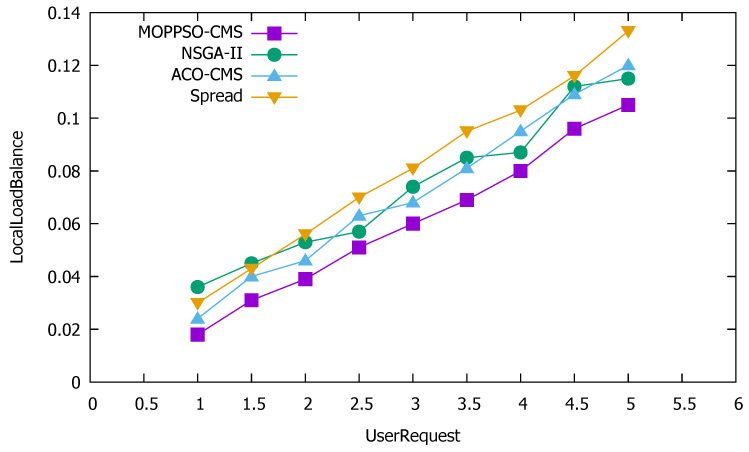
Performance differences of the four algorithms on local load balancing.

Local load balancing is an important indicator to measure the performance of the algorithm, which is also used in the scoring algorithm of the Kubernetes container scheduling system. In order to achieve balanced use of computing resources, storage resources, and memory resources, we considered local load balancing as an indicator. When a node focuses on a certain resource, a local load imbalance can occur. The smaller the value of this indicators, the better the local load balancing; while the larger the value is, the worse the local load balancing. Figure 10 shows the performance differences of the four algorithms, in terms of local load balancing.

Figure 10 shows that the Spread algorithm achieved reasonable performance under low user requests; however, when the amount of user requests increased, the performance worsened. This is because the algorithm tried to divide each container to each physical node and, when there are few user requests, each physical node is allocated less containers, which does not result in reduced performance, as the resources are not overwhelmed. Both the ACO-CMS algorithm and the GA-MOCA algorithm fluctuated, and their performance was not stable. This may be because both algorithms optimize the load balancing of the cluster, but ignore local load balancing. The MOPPSO-CMS algorithm had the best effect, as it was specially designed to optimize the local load balancing, such that the resources on the physical nodes can be balanced and reasonably used in the calculation process.

#### 5.4.3. Standard Deviation of Cluster Resources

**Figure 11:**
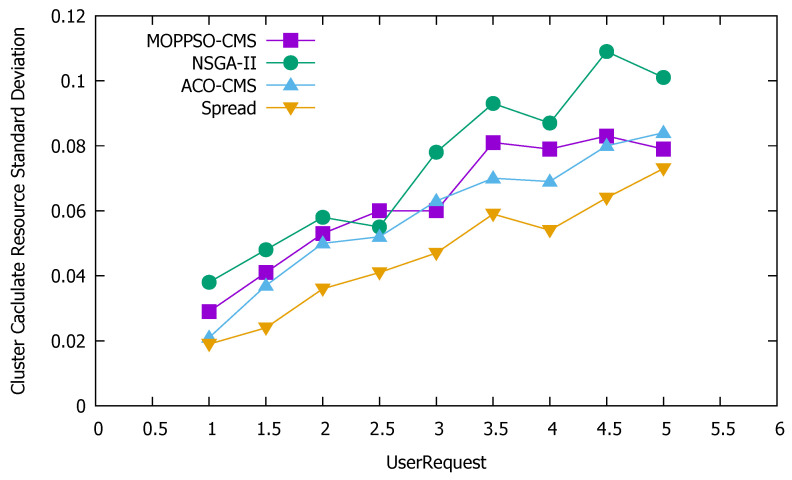
Standard deviation of the use of cluster computing resources.

**Figure 12:**
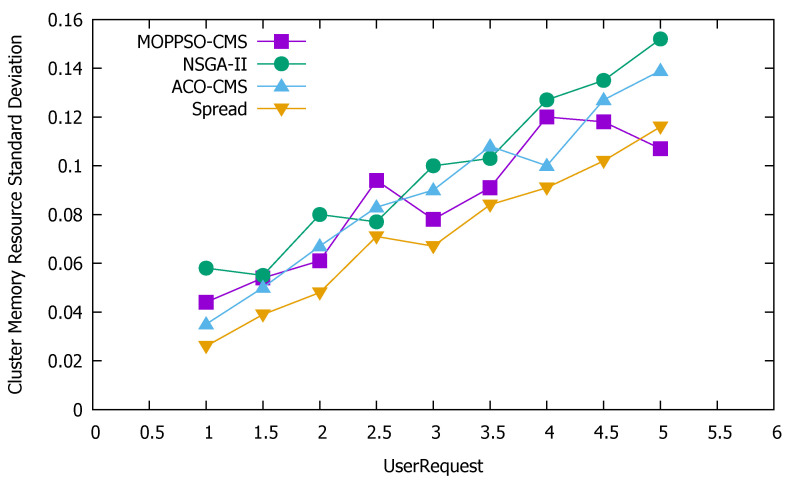
Standard deviation of the use of cluster memory resources.

**Figure 13:**
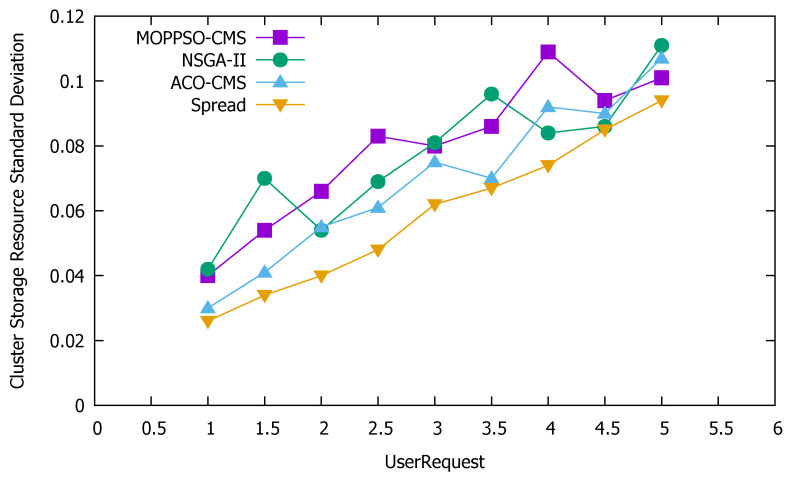
Standard deviation of the use of cluster storage resources.

Standard deviation is the most commonly used measure of statistical dispersion in probability statistics. It reflects the discrete degree between individuals within the group. According to this feature, we used the standard deviation to measure the discrete degree of node resource use in the cluster: the greater the value, the more discrete the value, while the smaller the value, the stabler it is. Figure 11 shows the standard deviation of the use of cluster computing resources, Figure 12 shows the standard deviation of the use of cluster memory resources, and Figure 13 shows the standard deviation of the use of cluster storage resources.

These figures indicate that the Spread algorithm maintained acceptable performance in the use of computing resources, storage resources, and memory resources in the experiment. This performance stems from the characteristics of the algorithm itself, which distributes all containers to each physical node as evenly as possible and achieves good results even without special optimization of the use of resources. The ACO-CMS algorithm also had good performance, as the container instance of the same microservice on a physical node is unique and the container allocation is relatively average, slightly better than the MOPPSO-CMS algorithm in this paper. The MOPPSO-CMS and NSGA-II algorithms in this paper do not have the same constraints as the ACO-CMS and Spread algorithms, such that multiple related containers can be allocated together to reduce the transmission overhead in the optimization; however, the load balancing is sacrificed. As we placed emphasis on local load balancing, the MOPPSO-CMS algorithm in this paper was superior to the NSGA-II algorithm, in most cases.

#### 5.4.4. Global Load Balancing

**Figure 14:**
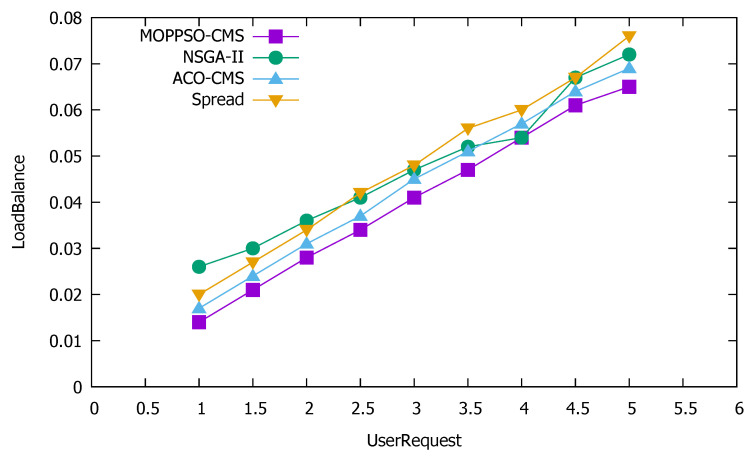
Performance differences of the four algorithms in global load balancing.

Global load balancing is an important index for measuring the resource usage of a cluster, which comprehensively considers the usage of cluster computing resources, storage resources, and memory resources. Based on traditional cluster load balancing, we combined local load balancing to achieve a more reasonable allocation of containers on physical nodes. The higher the value of this indicator, the heavier the cluster load, while the lower the value, the lighter the cluster load. Figure 14 shows the performance differences of the four algorithms in global load balancing.

Figure 14 shows that the Spread algorithm achieved good performance results when the number of requests was low, but the performance worsened when there was an increase in the number of requests, which also why this algorithm achieved good performance in local load balancing. The algorithm tries to distribute the containers to each physical node, and so it had a positive effect on the load balance when the number of requests was low. Both GA-MOCA and ACO-CMS optimize the load balancing of clusters, such that the performance of these two algorithms was better than that of the Spread algorithm; however, the ACO-CMS algorithm increased the optimization of storage usage on the basis of the GA-MOCA algorithm, so the performance of this algorithm was better than that of GA-MOCA. The above three algorithms ignored the optimization of memory usage and local load balancing in the cluster, factors which are within the optimization range of the MOPPSO-CMS algorithm, so the MOPPSO-CMS algorithm had better performance in cluster load balancing, compared to all other algorithms tested.

#### 5.4.5. Service Reliability

**Figure 15:**
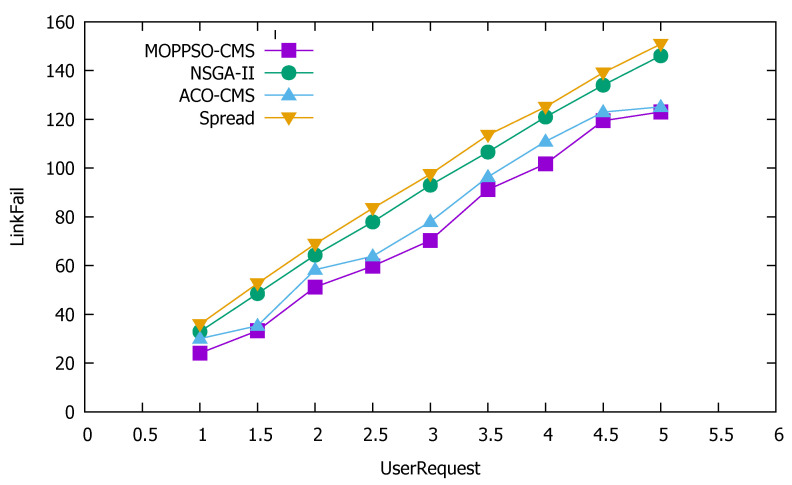
Performance comparison results of the three algorithms in service reliability.

Service reliability means the ability or possibility of a service to perform specified functions without fault within a certain time and under certain conditions. In order to be able to reasonably allocate containers, we use the number of requests that may fail as an indicator of service reliability. The lower the value, the more reliable the service, while the higher the value, the more unreliable the service. Figure 15 shows the performance comparison results of the three algorithms in service reliability.

Figure 15 shows that the Spread algorithm was the worst, as it divides the containers as evenly as possible between each physical node, which means that the number of requests that may fail during transmission between containers on different physical nodes can increase significantly. Although the ACO-CMS and GA-MOCA algorithms optimize service reliability, they still have shortcomings. The MOPPSO-CMS algorithm in this paper performed best, as it optimizes the requests between different physical nodes and between the same physical nodes, while the three other algorithms do not.

#### 5.4.6. Running Time

**Figure 16:**
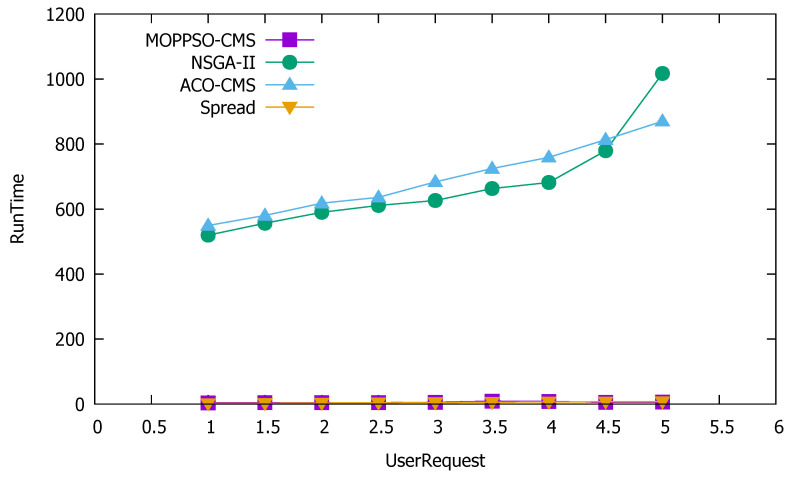
Performance comparison results of the four algorithms, in terms of running time.

Time complexity is an important indicator to measure the performance of an algorithm. The most intuitive manifestation is the time required for the algorithm to run the same data in the same environment; therefore, we took the running time of the algorithm in the experimental process as an indicator for time complexity. In this paper, the lower the running time of the algorithm, the better the performance of the algorithm. Table 6 and Figure 16 show the performance comparison of the four algorithms, in terms of running time. The running time was obtained using the time function in Python.

Table 6 shows that the running times of the MOPPSO-CMS and Spread algorithms were significantly lower than that of the GA-MOCA and ACO-CMS algorithms, which are shown in bold. The first reason is that the convergence rates of the NSGA and ACO algorithms are slow. The second reason is that the design of each ant and genome significantly increases the calculation time of the algorithm, when there are multiple containers present. The MOPPSO-CMS algorithm in this paper optimizes the shortcomings of the above two algorithms and uses the particle swarm optimization algorithm with faster convergence in parallel, which resulted in a quicker running time. The Spread algorithm achieves good performance in running speed, due to the algorithm’s simplicity.

## 6. Conclusions

In this paper, according to the characteristics of container microservice scheduling, three optimization objectives were proposed to reduce network transmission overhead, stabilize load balancing, and increase service reliability. A multi-objective optimization model was established, and a multi-objective optimization algorithm, based on the particle swarm optimization algorithm, was proposed to solve the microservice container scheduling problem. In this paper, parallel computing was used to ensure that different particle swarms share the optimal solution through inter-process interaction, which effectively avoids the particle swarm optimization algorithm falling into local optima. Further, we optimized the representation of particles, and successfully reduced the calculation time of the algorithm when multiple containers are present, addressing a critical disadvantage of the previously discussed methods. Compared to other algorithms, although our algorithm was slightly worse in partial load balancing than other algorithms, it had obvious advantages in reducing network transmission overhead, balancing cluster, node load balancing, improving service reliability, and reducing operation time. In future research, we plan to study the results of the proposed optimization algorithm in an actual physical cluster. On the basis of the three optimization objectives proposed in this paper, we will consider other optimization objectives, and try to combine other optimization algorithms with microservice container scheduling. Finally, future research will also include multi-objective optimization algorithm performance improvement methods.

## Figures and Tables

**Table 1:** Parameters relevant to the models discussed in this paper.

Element	Parameter	Description
Application	MS_SET	Microservice set of an application
	|MS_SET|=m	Total number of microservices
	MS_RELATION	Consumption relationship between
		microservices
Microservice	$ms_{i}\in MS\_SET$	Microservice *i*
	$Calc\_Req_{i}$	Computing resources required by a
		container of microservice *i*
	$Str\_Req_{i}$	Storage resources required for a
		container of microservice *i*
	$Mem\_Req_{i}$	Memory resources required for a
		container of microservice *i*
	$Fail_{i}$	Failure rate of container
	$CONS\_SET_{i}$	Microservice set consumed by
		microservice *i*
	$Link\_Thr_{i}$	The request threshold that can be processed by a
		container of microservice *i*
	$Link_{i}$	Total requests received for
		microservice *i*
	$Scale_{i}$	The number of containers of
		microservice *i* in the cluster
	$(ms_{i},ms_{l})\in$	Consumption relationship between
	MS_RELATION	microservice *i* and microservice *l*
	$Link(ms_{i},ms_{l})$	Total requests from microservice *i*
		to microservice *l*
	$Trans(ms_{i},ms_{l})$	Data transmission from microservice *i*
		to microservice *l*
Physical Node	$pm_{j}\in CLUSTER$	Physical node *j*
	|CLUSTER|=n	Total number of physical nodes
	$Calc\_Res_{j}$	Computing resources of physical nodes
	$Str\_Res_{j}$	Storage resources of physical nodes
	$Mem\_Res_{j}$	Memory resources of physical nodes
	$Fail_{j}$	Failure rate of physical nodes
Network	$Dist(pm_{j},pm_{j^{\prime }})$	Network distance between physical nodes
	$PassTime(pm_{j},pm_{j^{\prime }})$	Time required to transfer data between
		physical nodes

**Table 2:** MS_RELATION × 1.0 times of UserRequest.

(${ms}_{i},{ms}_{l}$)	${Link}_{i,j}$	${Trans}_{i,j}$	(${ms}_{i},{ms}_{j}$)	${Link}_{i,j}$	${Trans}_{i,j}$
(0, $ms_{1}$)	50	0	($ms_{7},ms_{14}$)	10	4.1
(0, $ms_{3}$)	70	0	($ms_{8},ms_{14}$)	15	4.2
(0, $ms_{6}$)	8	0	($ms_{9},ms_{5}$)	20	3.6
(0, $ms_{7}$)	30	0	($ms_{9},ms_{11}$)	20	4.7
(0, $ms_{10}$)	100	0	($ms_{10},ms_{5}$)	20	3.4
(0, $ms_{13}$)	30	0	($ms_{10},ms_{9}$)	25	4.4
($ms_{1},ms_{2}$)	20	4.6	($ms_{10},ms_{11}$)	20	4.9
($ms_{1},ms_{4}$)	10	3.1	($ms_{11},ms_{2}$)	20	3.2
($ms_{1},ms_{9}$)	20	4.0	($ms_{12},ms_{8}$)	45	6.4
($ms_{2},ms_{4}$)	10	3.5	($ms_{13},ms_{2}$)	20	4.5
($ms_{2},ms_{12}$)	15	5.9	($ms_{13},ms_{8}$)	45	6.1
($ms_{3},ms_{13}$)	60	1.8	($ms_{13},ms_{16}$)	8	5.5
($ms_{4},ms_{15}$)	30	5.6	($ms_{13},ms_{17}$)	30	2.4
($ms_{4},ms_{16}$)	8	5.7	($ms_{15},ms_{16}$)	8	5.2
($ms_{5},ms_{15}$)	30	5.3	($ms_{16},ms_{14}$)	15	4.3
($ms_{7},ms_{2}$)	20	4.8	($ms_{17},ms_{12}$)	15	6.2

**Table 3:** Microservices in application × 1.0 times of UserRequest.

${ms}_{i}$	CONS	${Cal}_{i}$	${Str}_{i}$	${Mem}_{i}$	${Scale}_{i}$	${Link}_{i}$	$Link\_{Thr}_{i}$	${Fail}_{i}$
1	2, 4, 9	2.1	1.4	2	5	10	50	0.04
2	4, 12	0.5	3.2	4	10	8	80	0.02
3	13	3.1	1.6	2	9	8	70	0.02
4	15, 16	4.7	0.2	2	4	5	20	0.02
5	15	1.8	3.1	2	10	8	40	0.002
6		2.5	5.1	2	2	4	8	0.02
7	2, 14	6.2	0.6	2	8	4	30	0.001
8	14	0.8	6.2	2	23	4	90	0.003
9	5, 11	3.9	2.3	2	9	5	45	0.001
10	5, 9, 11	0.2	4.8	4	25	4	100	0.006
11	2	2.8	2.6	2	5	8	40	0.02
12	8	5.3	0.9	2	8	4	30	0.003
13	2, 8, 16, 17	0.6	4.8	4	18	5	90	0.04
14		6.1	2.5	2	10	4	40	0.006
15	16	1.2	4.2	4	12	5	60	0.003
16	14	5.4	1.6	2	6	4	24	0.02
17	12	3.7	2.2	4	5	6	30	0.004

**Table 4:** Performance comparison of the algorithms with different parameters.

Userrequest	1	1.5	2	2.5	3	3.5	4	4.5	5
$pnum=100,iter\_num=100,c_{1}=0.2,c_{2}=0.2,\omega =0.2$									
fitness	29,749	67,121	107,454	173,738	222,159	286,247	353,762	403,526	493,399
$pnum=100,iter\_num=200,c_{1}=0.2,c_{2}=0.2,\omega =0.2$									
fitness	29,085	64,198	111,659	160,516	233,996	285,488	348,387	441,235	507,528
$pnum=100,iter\_num=300,c_{1}=0.2,c_{2}=0.2,\omega =0.2$									
fitness	29,885	62,481	107,957	155,796	217,289	278,558	352,253	412,551	496,465
$pnum=100,iter\_num=400,c_{1}=0.2,c_{2}=0.2,\omega =0.2$									
fitness	29,071	61,045	106,934	153,975	211,867	286,196	345,237	405,551	495,709
$pnum=200,iter\_num=100,c_{1}=0.2,c_{2}=0.2,\omega =0.2$									
fitness	29,633	63,695	106,443	159,145	224,563	276,847	351,607	418,323	508,780
$pnum=300,iter\_num=100,c_{1}=0.2,c_{2}=0.2,\omega =0.2$									
fitness	29,149	60,696	107,810	156,193	227,535	282,344	337,411	422,690	504,144
$pnum=400,iter\_num=100,c_{1}=0.2,c_{2}=0.2,\omega =0.2$									
fitness	29,541	63,340	107,729	156,117	215,572	270,340	334,728	426,384	473,625
$pnum=100,iter\_num=100,c_{1}=0.33,c_{2}=0.33,\omega =0.33$									
fitness	28,212	63,837	107,703	160,831	215,973	284,671	343,207	426,018	490,629
$pnum=100,iter\_num=100,c_{1}=0.2,c_{2}=0.2,\omega =0.6$									
fitness	29,306	63,346	110,912	160,118	210,481	282,464	366,727	415,715	511,287
$pnum=100,iter\_num=100,c_{1}=0.2,c_{2}=0.2,\omega =0.8$									
fitness	29,250	64,786	110,820	164,612	226,917	297,486	357,868	433,706	516,956
$pnum=100,iter\_num=100,c_{1}=0.2,c_{2}=0.6,\omega =0.2$									
fitness	26,232	58,685	97,800	145,278	202,630	267,078	344,999	405,686	463,625
$pnum=100,iter\_num=100,c_{1}=0.2,c_{2}=0.8,\omega =0.2$									
fitness	27,363	57,953	100,421	150,958	209,213	265,678	319,939	394,723	467,584
$pnum=100,iter\_num=100,c_{1}=0.6,c_{2}=0.2,\omega =0.2$									
fitness	31,289	70,993	118,831	171,354	231,513	289,272	366,174	463,794	522,502
$pnum=300,iter\_num=300,c_{1}=0.1,c_{2}=0.45,\omega =0.45$									
fitness	**25,742**	58,791	97,910	**139,280**	**192,893**	**250,241**	**304,531**	366,548	**438,265**
$pnum=300,iter\_num=300,c_{1}=0.1,c_{2}=0.6,\omega =0.3$									
fitness	26,768	**55,467**	**96,711**	143,826	200,094	251,660	309,181	**365,054**	454,318

**Table 5:** Relevant parameters of the MOPPSO-CMS algorithm.

pnum	iter_num	ω	$c_{1}$	$c_{2}$
300	300	0.45	0.1	0.45

**Table 6:** Performance comparison of the four algorithms, in terms of running time(second).

UserRequest	MOPPSO-CMS	GA-MOCA	ACO-CMS	Spread
1	**3.90**	519.58	549.34	**1.72**
1.5	**4.34**	556.22	580.15	**2.52**
2	**4.16**	589.94	617.91	**3.33**
2.5	**4.26**	611.03	636.04	**4.20**
3	**5.71**	626.36	683.64	**4.88**
3.5	**8.86**	663.00	724.85	**5.93**
4	**7.90**	681.74	759.16	**6.56**
4.5	**5.70**	779.46	813.50	**7.24**
5	**6.06**	1017.02	870.25	**8.13**

## Data Availability

Not applicable.
